# Identification and Analysis of Chemical Constituents and Rat Serum Metabolites in Gushuling Using UPLC-Q-TOF/MS Coupled with Novel Informatics UNIFI Platform

**DOI:** 10.1155/2021/2894306

**Published:** 2021-12-31

**Authors:** Hong Chang, Shujie Lv, Tengteng Yuan, Huan Wu, Lei Wang, Ran Sang, Caiyun Zhang, Weidong Chen

**Affiliations:** ^1^College of Pharmacy, Anhui University of Chinese Medicine, Hefei 230012, China; ^2^The Experiment Research Center, Anhui University of Chinese Medicine, Hefei 230012, China; ^3^Anhui Province Key Laboratory of Chinese Medicinal Formula, Hefei 230012, China; ^4^Bengbu Medical College, Bengbu 233030, China; ^5^The First Affiliated Hospital of Bengbu Medical College, Bengbu 233004, China

## Abstract

Gushuling (GSL), a well-known hospital preparation composed of traditional Chinese medicine (TCM), has been widely used in the clinical treatment of osteoporosis (OP) for decades due to its remarkable therapeutic effect. However, the chemical constituents of GSL are still unclear so far, which limits the in-depth study of its pharmacodynamic material basis and further restricts its clinical application. In this study, we developed a strategy for qualitative analysis of the chemical constituents of GSL *in vitro* and *in vivo*. Based on the results of ultra-performance liquid chromatography coupled with quadrupole time-of-flight tandem mass spectrometry (UPLC-Q-TOF-MS) and the UNIFI informatics platform, the chemical constituents of GSL can be determined quickly and effectively. By comparing the retention time, accurate mass, and fragmentation spectrum of the compounds in GSL, a total of 93 compounds were identified or preliminarily identified, including flavonoids, terpenoids, phenylpropanoids, steroids, etc. Among them, nine compounds have been confirmed by standard substances, namely epimedin A, epimedin B, epimedin C, icariin, ecdysterone, calycosin, calycosin-7-glucoside, ononin, and ginsenoside Ro. Fragment patterns and characteristic ions of representative compounds with different chemical structure types were analyzed. At the same time, 20 prototype compounds and 42 metabolites were detected in rat serum. Oxidation, hydration, reduction, dehydration, glutathione S-conjugation, and acetylcysteine conjugation were the main transformation reactions of GSL in rat serum. In this research, the rapid method to characterize the *in vitro* and *in vivo* chemical constituents of GSL can not only be used for the standardization and quality control of GSL but also be helpful for further research on its pharmacodynamic material basis.

## 1. Introduction

Osteoporosis (OP) is a complex bone disease characterized by low bone density and impaired microstructure of bone tissue, leading to increased bone fragility and being prone to fractures [1]. At present, the treatment drugs for OP mainly include ingredients that inhibit bone resorption, such as bisphosphonates, calcitonin, and estrogen, and ingredients that promote bone formation, such as parathyroid hormone [2]. However, taking bisphosphonates for a long time will increase the incidence of complications such as osteonecrosis of the jaw [3,4]. Long-term use of estrogen will also increase the incidence of breast cancer, endometrial cancer, and other diseases [5,6]. Nowadays, the importance of traditional Chinese medicine (TCM) and Chinese herbal compound prescription that have a therapeutic effect on OP were gradually been recognized because of their safety and effectiveness [7–9].

Gushuling (GSL) is a TCM preparation in the hospital of the First Affiliated Hospital of Anhui University of Chinese Medicine, consisting of *Herba Epimedii* (Yinyanghuo), *Radix Achyranthis Bidentatae* (Niuxi), *Radix Astragali* (Huangqi), and *Concha Ostreae* (Muli) as recorded in the Chinese Pharmacopoeia (Table S1). GSL has a good effect on the management of various types of OP in clinical treatment, such as senile osteoporosis [10,11], postmenopausal osteoporosis [12], and diabetes secondary osteoporosis [13]. However, the chemical constituents of TCM and related preparations are complex and the active ingredients are not fully understood [14,15]. Although the chemical constituents of GSL have been previously reported, only two or three bioactive ingredients have been found in GSL [16,17]. These efforts failed to reflect the overall chemical constituents of GSL, which made it was difficult to comprehensively evaluate the quality of GSL. Therefore, there is an urgent need for a reliable and efficient analytical method to determine the chemical constituents of GSL and the effective ingredients that can enter the body to achieve the purpose of quality control. Fortunately, the combination of ultra-high-performance liquid chromatography coupled with quadrupole time-of-flight tandem mass spectrometry (UPLC-Q-TOF-MS) and the UINIF analysis platform can solve this problem to some extent.

In recent years, UPLC-Q-TOF-MS has been widely used in various fields such as qualitative analysis of TCM ingredients, serum pharmacochemistry, metabolomics, and new drug development due to its high resolution and high sensitivity [18–21]. As a powerful information processing platform, UNIFI can automatically extract the mass spectrometry data of the sample, determine the molecular formula, compare it with the compound in the database, which gives the possible cracking method of the compound according to the fragment information of the compound under high energy, and display the detailed information of the identified compound under the preset filter conditions [22,23]. The purpose of this study was to use the UPLC-Q-TOF-MS technology in conjunction with UNIFI analysis software to comprehensively analyze and identify the chemical constituents of hospital preparation GSL and to analyze the prototype compounds and metabolites in rat serum, which provides a basis for the in-depth research and quality control of its pharmacodynamic material basis.

## 2. Materials and Methods

### 2.1. Materials and Reagents

*Herba Epimedii*, *Radix Achyranthis Bidentatae*, *Radix Astragali*, and *Concha Ostreae* were purchased from Anhui Xiehecheng Co., Ltd. (Bozhou, China) and evaluated by Doctor Rongchun Han (College of Pharmacy, Anhui University of Chinese Medicine, Hefei, China). The standard substances of calycosin (Lot number: MUST-18120901), calycosin-7-glucoside (Lot number: MUST-19031920), ononin (Lot number: MUST-19041101), epimedin C (Lot number: MUST-19081310), icariin (Lot number: MUST-18091010), and ecdysterone (Lot number: MUST-18120209) were purchased from Chengdu Must Bio-Technology Co., Ltd. (Chengdu, China). Epimedin A (Lot number: JOT-10354), epimedin B (Lot number: JOT-10353), and ginsenoside Ro (Lot number: JOT-10448) were purchased from Chengdu Pufei De Biotech Co., Ltd. (Chengdu, China). The purity of all standards was over 98.0%. Acetonitrile (HPLC grade; Lot number: SHBM1775) and methanol were purchased from Sigma Aldrich Trading Co., Ltd. (Shanghai, China). Formic acid (HPLC grade; Lot number: J2027129) was obtained from Aladdin Biochemical Technology Co., Ltd. (Shanghai, China). Ultrapure water was prepared by a Milli Q-Plus system (Millipore, Bedford, USA).

### 2.2. Standards and Sample Preparation

First, *Herba Epimedii* and *Radix Achyranthis Bidentatae* were mixed at the ratio of 5 : 4 and refluxed with 70% ethanol at 8-fold volume (11-fold volume for the first time) for 3 times for 2 h, respectively. We used a rotary evaporator to volatilize the ethanol in the extract to obtain a concentrated solution for use. Second, *Radix Astragali* and *Concha Ostreae* were mixed at the ratio of 5 : 2 and boiled with 9.5-fold and 8-fold volume ultrapure water for 1 h, respectively. Finally, the ethanol extract obtained in the first step was mixed with the water extract obtained in the second step and concentrated to obtain the GSL sample solution. *Herba Epimedii* refluxed with 70% ethanol at 8-fold volume (11-fold volume for the first time) for 3 times for 2 h, respectively. We used a rotary evaporator to volatilize the ethanol and water in the extract to obtain a sample solution of *Herba Epimedii*. The preparation method of *Radix Achyranthis Bidentatae* sample solution is the same as that of *Herba Epimedii* sample solution. *Radix Astragali* boiled with 9.5-fold and 8-fold volume ultrapure water for 1 h, respectively, and concentrated to obtain the *Radix Astragali* sample solution. An appropriate amount of sample solution was diluted with methanol, and the supernatant was taken and stored at 4°C. All the solutions were filtered through a 0.22 *µ*m filter membrane before analysis.

Nine standard substances were completely dissolved in methanol. Before qualitative analysis, they were mixed to prepare a mixed standard solution with appropriate concentration and passed through a 0.22 *µ*m filter membrane. All solutions were stored in a refrigerator at 4°C.

### 2.3. Animals Handling and Serum Samples Preparation

Sprague Dawley rats (200 ± 20 g) of specific pathogen-free grade were purchased from Animal Experiment Center, Bengbu Medical College. Approved by the Experimental Animal Management and Ethics Committee of Bengbu Medical College, animal experimental research meets relevant ethical requirements. They were randomly divided into blank groups and GSL extract groups, with 6 rats in each group. The rats were kept in an animal room with a suitable environment for 7 days before the experiment. Rats in the blank group and GSL extract group were intragastrically administered distilled water and 51.993 g·kg^−1^·d^−1^ GSL sample solution for consecutive 3 days, respectively. The preparation process of the GSL sample solution is the same as that in the Standards and Sample Preparation section, and there is no need for subsequent operations such as methanol treatment. Before the last oral administration, the rats fasted for 12 h with free drinking water. Blood samples (500 *µ*L) were collected from the fundus venous plexus 15 min after oral administration on the 3rd day and centrifuged for 10 min at 3500 rpm·min^−1^ at 4°C, and the supernatant was taken. Then, 1200 *µ*L methanol was added to the 300 *µ*L serum samples, vortexed, and centrifuged at 13000 rpm for 10 min. The supernatant was put into another tube and dried with nitrogen gas. The remaining was stored in acetonitrile (200 *µ*L) and frozen at −80°C until analysis.

### 2.4. Chromatography and Mass Spectrometry Conditions

Chromatographic analysis was performed using a Waters Acquity™ UPLC system (Waters Corporation, Milford, USA). Chromatographic separation was carried on Waters ACQUITY UPLC^®^ BEH C18 (2.1 × 100 mm, 1.7 *µ*m) column by gradient elution with the optimal mobile phase of 0.1% formic acid aqueous solution (solvent A) and acetonitrile (solvent B), the column temperature was maintained at 35°C, and the temperature of the sample chamber was set to 8°C. The gradient elution was set as follows: 0−5 min, 5%–19% B; 5–10 min, 19% B; 10–11 min, 19%–25% B; 11–16 min, 25% B; 16–17 min, 25%–31% B; 17–22 min, 31%–51% B; 22–50 min, 51%–100% B; 50–55 min, 100% B; 55–60 min, 100%–5% B; 60–65 min, 5% B. The flow rate was 0.15 mL·min^−1^, and the injection volume was 2 *µ*L.

A Waters Xevo G2 Q-TOF mass spectrometer (Waters Corporation, Milford, USA) equipped with an electrospray ionization (ESI) source operating in both positive and negative ion modes was connected to the UPLC. The full scan data were collected from m/z 50 to m/z 1200. For positive and negative ion modes, the capillary and cone voltage were set to 3.0 kV, 40 V and 2.5 kV, 40 V, respectively. The temperature of the conservation gas was set to 350°C, and the flow rate was set to 600 L·h^−1^. The two ion source temperatures in positive ion and negative ion modes were set to 120°C and 110°C, respectively. The cone gas flow rate was set to 50 L· h^−1^, and leucine and enkephalin were used as calibration fluids to ensure accuracy and repeatability.

### 2.5. UNIFI Data Processing Method

The chemical constituents analysis strategy was mainly divided into the following three steps:

1. The establishment of the chemical constituents library of GSL: The complete information on the compounds of three herbal medicines (*Herba Epimedii*, *Radix Achyranthis Bidentatae*, and *Radix Astragali*) in GSL was collected and obtained by searching China National Knowledge Infrastructure (CNKI), PubMed, PubChem, Traditional Chinese Medicine Systems Pharmacology Database and Analysis Platform (TCMSP), ChemSpider, and other databases. The self-built compound library was established, including compound name, molecular formula, chemical structure (saved in “mol” format), accurate molecular mass, and other information. This information was imported into UNIFI. Among them, a total of 354 compounds were listed. When analyzing rat serum samples, the self-built database imported into UNIFI was the GSL *in vitro* compound information database that has been analyzed and confirmed.
2. Preliminary analysis of the results: We imported the original files of GSL sample solution, blank sample solution, rat administration serum, and blank serum analyzed by UPLC-Q-TOF-MS into the UNIFI software for samples comparison. Based on the automatic matching function of UNIFI software, compounds can be quickly identified. The parameter settings were as follows: analysis time range, 1–65 min; quality allowable error range, ±10 ppm; quality testing range, 50 Da to 1500 Da; positive adducts including H^+^, Na^+^, and K^+^; and negative adducts containing H^−^, HCOO^−^, and Cl^−^.
3. Manual review: By the MassLynx workstation, the above identification results were reviewed in combination with the precise mass of excimer ions, retention time, fragment ion information, and literature.

## 3. Results and Discussion

### 3.1. Chemical Constituents Analysis of Three Herbal Medicines in GSL

The separate extraction method of the three herbal medicines in GSL (*Herba Epimedii*, *Radix Achyranthis Bidentatae*, and *Radix Astragali*) was the same as the sample preparation process. Then, the TIC of the three herbal medicines in positive and negative ion modes were qualitatively analyzed according to the above-mentioned method, and the results are shown in Figure S1.

### 3.2. Identification and Analysis of Chemical Constituents in GSL

**Figure 1:**
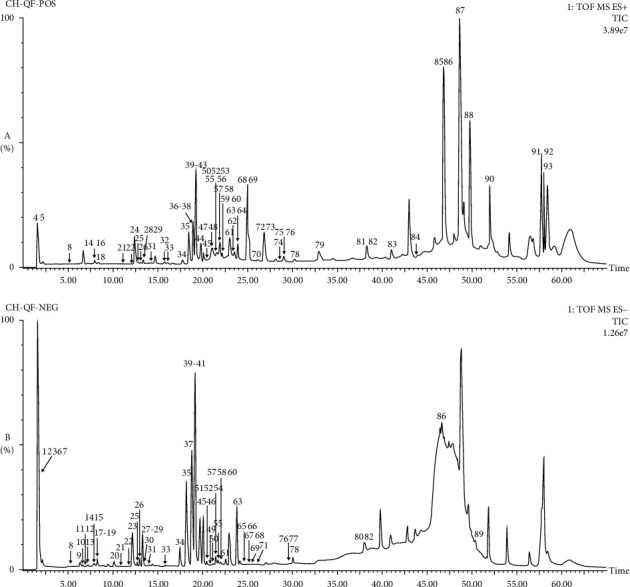
The total ion chromatography (TIC) of GSL from UPLC-Q-TOF-MS analysis. (a) Positive ion mode. (b) Negative ion mode.

**Figure 2:**
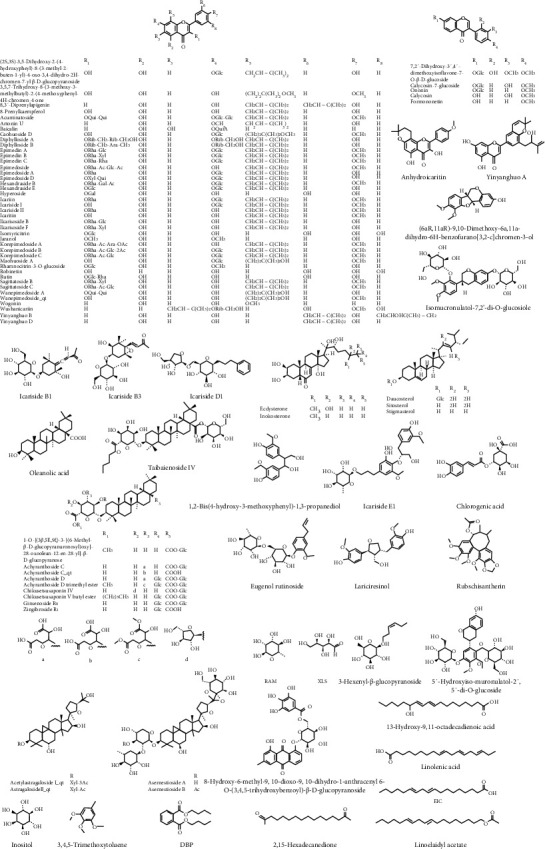
Chemical structures of compounds identified in GSL.

High-resolution mass spectrometry data of GSL were rapidly acquired by the UPLC-Q-TOF-MS method. The TIC of GSL in positive and negative ion modes is portrayed in Figure 1. The UNIFI screening platform was used to process and analyze its mass spectrometry data, and then the data were spontaneously matched with the fragment information. After further manual verification, it was found that there were 93 *in vitro* chemical constituents in the GSL recipe, including 51 kinds of flavonoids, 18 kinds of terpenoids, 6 kinds of phenylpropanoids, 5 kinds of steroids, and 13 others. Among them, 55 compounds were from *Herba Epimedii*, 23 compounds from *Radix Achyranthis Bidentatae*, and 21 compounds from *Radix Astragali*. In addition, *Herba Epimedii* and *Radix Achyranthis Bidentatae* contain four common compounds, and *Herba Epimedii*, *Radix Achyranthis Bidentatae*, and *Radix Astragali* contain one identical compound (rutin). Of the identified components, 9 compounds were identified by comparison with the standard substance. The detailed information on these components is gathered in Table 1 and Figure 2.

### 3.3. Identification and Analysis of Chemical Constituents in Rat Serum

When analyzing the rat serum samples of GSL, the selected database was a self-built database constructed from analyzed and confirmed GSL *in vitro* compound information. The analysis steps of GSL *in vivo* components were the same as *in vitro* processing. The results showed that there were 20 chemical constituents in rat serum, including10 kinds of flavonoids, 4 kinds of triterpenoids, 2 kinds of phenylpropanoids, 1 kind of steroid, 1 kind of organic acid, and 2 others. Among them, 12 compounds were from *Herba Epimedii*, 8 compounds from *Radix Achyranthis Bidentatae*, and 2 compounds from *Radix Astragali*. At the same time, rutin is a common compound of three Chinese herbal medicines. Five compounds were identified by the standard substance. The specific information is shown in Table 2 and Figure S2.

### 3.4. Identification and Analysis of Metabolites in Rat Serum

After the chemical constituents from the GSL get into the body, some components exist in the form of a prototype, but most of them are structurally modified based on the original components, such as oxidation, reduction, and hydration. With the unique data postprocessing function of UNIFI, we analyzed the possible metabolites based on the screened prototype compounds *in vivo* and finally obtained 42 metabolites in rat serum through artificial screening. After analysis, these 42 metabolites mainly undergo oxidation, hydration, reduction, dehydration, glutathione S-conjugation, and acetylcysteine conjugation reactions based on the prototype compounds. The detailed information of the prototype, metabolite name, molecular weight, and molecular formula is given in Table 3.

### 3.5. Analysis of GSL by UPLC-Q-TOF-MS

#### 3.5.1. Flavonoids

Flavonoids and their glycosides are the main ingredients and the major bioactive components of GSL. In this study, 3 flavones, 41 flavonols, 5 isoflavones, 1 isoflavanone, and 1 flavanol were determined by the matching of the mass spectrometry data with the UNIFI analysis platform.

For flavonoid glycosides, the glycosidic bonds connected by oxygen atoms could be cleaved in both positive and negative ion modes, and most of them were characterized by neutral losses such as 162 Da (Glc) and 146 Da (Rha) [24]. It was difficult to directly remove the glycosyl groups connected by carbon-glycosidic bonds and often produce [M+H−120]^+^ fragment [25]. As we all know, the main cleavage behavior of flavonoid aglycones was the retro Diels–Alder reaction (RDA) cleavage pathway and the loss of free radicals and/or small molecules (such as CH_3_, CO, and CO_2_) [26]. By comparing the retention time and fragmentation patterns with standard substance, peaks 14, 31, 33, 34, 35, 37, and 41 in Figure 1 were exactly identified as calycosin-7-glucoside, ononin, calycosin, epimedin A, epimedin B, epimedin C, and icariin, respectively. Here, we took epimedin C and icariin as examples to depict the fragmentation mode of these ingredients.

**Figure 3:**
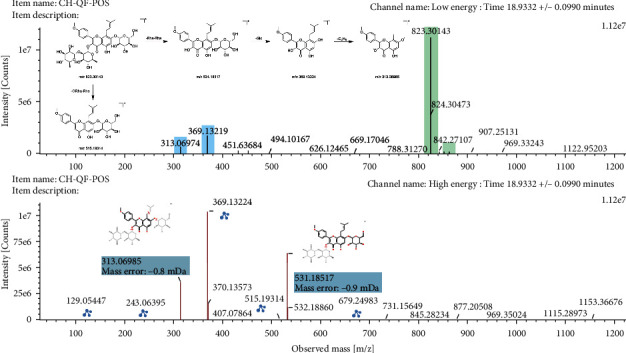
The mass spectrogram and fragmentation pathways of epimedin C in positive ion mode.

Epimedin C showed quasi-molecular ion [M+H]^+^ at m/*z* 823.3014 in positive ion mode and yielded fragment ions at m/*z* 531.1852 and 515.1931 by losses of 2 molecules of rhamnose and a molecule of ORha-Rha group, respectively. Then, the ion at m/*z* 515.1931 loses a molecule of Glc to generate an ion at m/*z* 369.1322, and the ion at m/*z* 369.1322 further loses a functional group of C_4_H_8_ to produce ion at m/*z* 313.0699. The mass spectrogram and possible fragmentation pathways of epimedin C in positive ion mode are shown in Figure 3.

In the positive ion mode, the mass-to-charge ratio of the quasi-molecular ion peak of compound 41 was 677.2433 [M+H]^+^ as shown in Table 1. The mass-to-charge ratio of the fragment ions produced by the precursor ions were 531.1851 [M+H−Rha]^+^, 369.1321 [M+H−Glc]^+^, and 313.0698 [M+H−C_4_H_8_]^+^, which are consistent with those of icariin. The mass spectrogram and potential fragmentation pathways of icariin in positive ion mode are shown in Figure S3.

#### 3.5.2. Terpenoids

The terpenoids in GSL primarily included monocyclic monoterpenoids, cycloartane-type tetracyclic triterpenoids, and oleanane-type pentacyclic triterpenoids. Among them, the number of the above categories in GSL was 3, 4, and 11, respectively.

**Figure 4:**
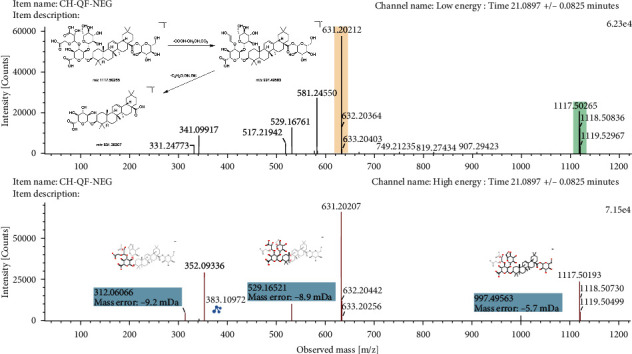
The mass spectrogram and fragmentation pathways of achyranthoside D in negative ion mode.

Triterpene saponins in GSL mainly exist in the form of aglycones binding with sugars, such as glucose, rhamnose, and xylose. In mass spectrometric analysis, triterpenoid saponins are mostly in the form of de-sugar or continuous de-sugar fragment ions [27]. Such compounds also had the loss of CO_2_, COOH, CH_2_OH, and other complex groups. Compound 49 gave a deprotonated molecule [M−H]^−^ at m/*z* 1117.5026 and produced predominant fragment ions at m/*z* 997.4956 [M−H−COOH−CH_2_OH−CO_2_]^−^ and m/*z* 631.2021 [M−H−C_2_H_2_O−2Glc]^−^ in negative ion mode (Figure 4). It was consistent with previous literature [28] and was finally identified as achyranthoside D. Peak 51 was identified clearly as ginsenoside Ro with a standard substance, and its mass fragmentation pattern is demonstrated in detail (Figure S4). In the negative ion mode, ginsenoside Ro gave [M−H]^−^ ion at m/*z* 955.4884, along with two major fragment ions at m/*z* 793.4236 [M−H−Glc]^−^ and 631.2021[M−H−2Glc]^−^ in mass spectrometry under high-energy conditions. From the cleavage pathway of these triterpenoid saponins, they tend to lose the sugar group at the C28 position first under the action of high energy of MS^E^. The possible reason for this phenomenon is that the ester bond at the C28 position is easier to break than the ether bond at the C3 position.

#### 3.5.3. Phenylpropanoids

**Figure 5:**
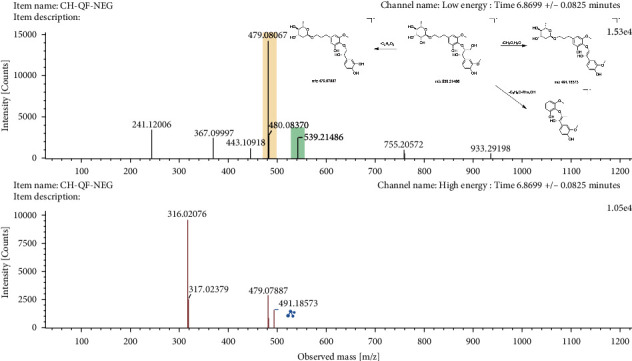
The mass spectrogram and fragmentation pathways of icariside E1 in negative ion mode.

Six phenylpropanoids were recognized as the major active ingredients in GSL. Among them, a total of 2 phenylpropionic acids, 1 phenylpropanol, 1 styrene, and 2 lignans were identified. Simple phenylpropanoids belong to phenylpropane derivatives in structure and exist in plants in the form of glycosides or esters, which can be combined with sugars and polyols. In mass spectrometric analysis, phenylpropanoids are mainly manifested by the loss of sugar, neutral loss, and loss of other complex groups [29]. Compound 11 showed a deprotonated molecule [M−H]^−^ at m/*z* 539.2149 and produced predominant fragment ions at m/*z* 491.1857 [M−H−CH_2_O−H_2_O]^−^, m/*z* 479.0789 [M−H−C_2_H_4_O_2_]^−^, and m/*z* 317.0238 [M−H−C_3_H_6_O−Rha−OH]^−^ in negative ion mode. By confirmation of fragment ions, we preliminarily identified compound 11 as icariside E1. In the case of compound 44, it generated a base peak ion at m/*z* 481.1785 [M+Na]^+^ in positive ion mode, along with a major fragment ion at m/*z* 387.1427 [M+Na−C_4_H_8_O]^+^, which was consistent with a previous study [30]. Finally, it was assigned to be rubschisantherin. The detailed mass spectrogram and fragmentation pathways are shown in Figures 5 and S5.

#### 3.5.4. Steroids

**Figure 6:**
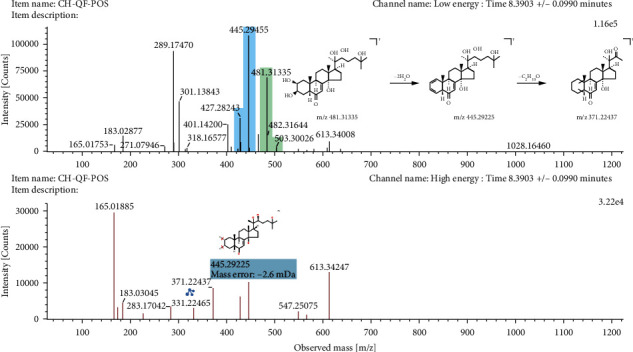
The mass spectrogram and fragmentation pathways of ecdysterone in positive ion mode.

In the UNIFI results interface, 5 steroids were automatically matched. The cleavage of steroids and their aglycones is complicated. Besides RDA cleavage, dehydration, and demethylation of the hydroxyl group, the side chain at position 17 often falls off. Peak 18 was ascertained to be ecdysterone by contrast with reference standards. As shown in Figure 6, ecdysterone displayed a hydrogenated ion at m/*z* 481.3134 [M+H]^+^ with a molecular formula C_27_H_44_O_7_ and lost H_2_O to generate an ion at m/*z* 445.2923 [M+H−2H_2_O]^+^. Further loss of the C_4_H_10_O group resulted in fragmentation with a m/*z* of 371.2244 [M+H−2H_2_O−C_4_H_10_O]^+^.

#### 3.5.5. Others

Some compounds with fewer species and lower concentrations are assigned to this category. The mass spectra data extracted from MassLynx workstation were matched with UNIFI software, and the results were verified by literature analysis [31]. A total of 13 compounds were inferred, including anthraquinones, glycosides, organic acids, and others. Specific mass spectrometry data are listed in Table 1.

## 4. Conclusions

In this experiment, UPLC-Q-TOF-MS technology combined with UNIFI software was used for the first time to comprehensively and systematically analyze the *in vitro* and *in vivo* chemical constituents of GSL. We summarized the cleavage law of flavonoids, triterpene saponins, phenylpropanoids, and steroids in the mass spectrum and initially explored the prototype compounds and metabolites of GSL in rat serum. These results provide a technical basis for the comprehensive and effective quality control and pharmacodynamic material basis of GSL. In addition, some chromatographic peaks with better response in GSL are unknown ingredients, which deserve further study.

## Figures and Tables

**Table 1:** Identification of chemical constituents of GSL by UPLC-Q-TOF-MS.

NO.	Observed RT (min)	Formula	Observed m/z	Mass error (ppm)	Adducts	Fragment (+/−)	Identification	Structure class	Origin
1	1.63	C_6_H_12_O_5_	209.0670	1.3	[M+HCOO]^−^	89.0255	RAM	Saccharide	HQ
2	1.64	C_5_H_10_O_5_	195.0510	−0.2	[M+HCOO]^−^	89.0255	XLS	Saccharide	HQ
3	1.66	C_23_H_24_O_11_	475.1287	8.6	[M-H]^−^	89.0255, 179.0563, 290.0864, 341.1084	7,2′-Dihydroxy-3′,4′-dimethoxyisoflavone-7-O-*β*-D-glucoside	Flavonoids	HQ
4	1.67	C_6_H_12_O_6_	203.0522	−2.2	[M+Na]^+^, [M+K]^+^	127.0388	Inositol	Others	YYH
5	1.68	C_10_H_14_O_3_	205.0836	0.4	[M+Na]^+^	127.0388	3,4,5-Trimethoxytoluene	Others	YYH
6	1.69	C_27_H_32_O_12_	593.1879	0.6	[M+HCOO]^−^	89.0255, 179.0563, 290.0864, 341.1084, 377.0850	Maohuoside A	Flavonoids	YYH
7	1.76	C_21_H_22_O_7_	431.1381	7.8	[M+HCOO]^−^	128.0362	Wanepimedoside_qt	Flavonoids	YYH
8	5.25	C_16_H_18_O_9_	353.0867	−3.1	[M−H]^−^	191.0556	Chlorogenic acid	Phenylpropanoids (phenylpropionic acid)	YYH
		C_16_H_18_O_9_	355.1041	4.9	[M+H]^+^, [M+Na]^+^	163.0387	Chlorogenic acid	Phenylpropanoids (phenylpropionic acid)	YYH
9	6.44	C_19_H_30_O_8_	431.189	−6.6	[M+HCOO]^−^	163.0409, 173.0463, 219.0629	Icariside B1	Terpenoids (monocyclic monoterpenoids)	YYH
10	6.49	C_19_H_28_O_10_	461.1704	8.6	[M+HCOO]^−^	219.0629	Icariside D1	Terpenoids (monocyclic monoterpenoids)	YYH
11	6.87	C_26_H_36_O_12_	539.2149	2.7	[M−H]^−^	317.0238, 479.0789, 491.1857	Icariside E1	Phenylpropanoids (phenylpropanols)	YYH
12	6.92	C_21_H_20_O_13_	479.0807	−5.1	[M−H]^−^	316.0208	Isomyricitrin	Flavonoids	YYH
13	7.22	C_32_H_38_O_15_	697.1844	−8.8	[M+Cl]^−^	191.0563, 219.0647, 593.1515	Ikarisoside B	Flavonoids	YYH
14	7.86	C_22_H_22_O_10_	491.1183	−2.5	[M+HCOO]^−^	283.0601	Calycosin-7-glucoside^*∗*^	Flavonoids	HQ
		C_22_H_22_O_10_	447.1279	−1.4	[M+H]^+^	285.0749	Calycosin-7-glucoside^*∗*^	Flavonoids	HQ
15	7.92	C_21_H_20_O_12_	463.0849	−7.1	[M−H]^−^	285.0396, 431.0962	Hyperoside	Flavonoids	YYH NX
16	8.00	C_15_H_10_O_7_	303.0520	6.8	[M+H]^+^	287.0543	Robinetin	Flavonoids	YYH
17	8.17	C_28_H_34_O_12_	597.1798	9.1	[M+Cl]^−^	193.0869, 219.0659, 237.0750, 399.1276	Caohuoside D	Flavonoids	YYH
18	8.39	C_27_H_44_O_7_	525.3065	−0.8	[M+HCOO]^−^, [M+Cl]^−^	159.1057, 193.0869, 219.0659	Ecdysterone^*∗*^	Steroids	NX
		C_27_H_44_O_7_	481.3134	−5.5	[M+K]^+^, [M+Na]^+^	371.2244, 445.2923	Ecdysterone^*∗*^	Steroids	NX
19	8.42	C_22_H_32_O_11_	517.1889	−7.2	[M+HCOO]^−^, [M−H]^−^, [M+Cl]^−^	177.0907, 471.1833	Eugenol rutinoside	Phenylpropanoids (Styrene)	YYH
20	10.13	C_29_H_38_O_15_	625.2131	−1.1	[M−H]^−^	261.0762, 279.0868, 291.0852	Isomucronulatol-7,2′-di-O-glucosiole	Flavonoids	HQ
21	11.14	C_16_H_12_O_5_	283.0599	−4.7	[M−H]^−^	268.0381	Wogonin	Flavonoids	NX
		C_16_H_12_O_5_	285.0769	4.0	[M+H]^+^	270.0515	Wogonin	Flavonoids	NX
22	12.07	C_32_H_38_O_16_	677.2074	−1.9	[M−H]^−^	353.0993, 514.1457, 515.1522, 557.1635	Hexandraside E	Flavonoids	YYH
		C_32_H_38_O_16_	679.2231	−0.3	[M+H]^+^, [M+Na]^+^	299.0524, 355.1186, 517.1787	Hexandraside E	Flavonoids	YYH
23	12.18	C_43_H_68_O_14_	853.4619	3.3	[M+HCOO]^−^, [M−H]^−^	96.9603, 451.3268	1-O-{(3*β*,5*ξ*,9*ξ*)-3-[(6-Methyl-*β*-D-glucopyranuronosyl)oxy]-28-oxoolean-12-en-28-yl}-*β*-D-glucopyranose	Triterpenoids	NX
24	12.41	C_29_H_50_O	453.3471	−4.8	[M+K]^+^	343.3055	Sitosterol	Steroids	YYH NX
25	12.81	C_38_H_48_O_20_	825.2805	−0.8	[M+H]^+^	299.0541, 355.1165, 517.1669, 663.2131	Diphylloside A	Flavonoids	YYH
		C_38_H_48_O_20_	869.2693	−3.2	[M+HCOO]^−^, [M−H]^−^, [M+Cl]^−^	146.9657, 353.1024, 514.1468, 661.2136, 807.2701	Diphylloside A	Flavonoids	YYH
26	13.06	C_37_H_46_O_19_	793.2564	0.4	[M−H]^−^, [M+Cl]^−^, [M+HCOO]^−^	285.0389, 431.0974, 631.2028	Epimedoside D	Flavonoids	YYH
		C_37_H_46_O_19_	795.2681	−3.2	[M+H]^+^	287.0541, 299.0538, 355.1168, 517.1700	Epimedoside D	Flavonoids	YYH
27	13.29	C_37_H_44_O_17_	805.2492	−8.5	[M+HCOO]^−^	146.9657, 353.1024, 514.1468, 661.2136	Epimedoside	Flavonoids	YYH
28	13.32	C_38_H_48_O_19_	807.2703	−1.8	[M−H]^−^	146.9657, 353.1024, 514.1468, 661.2136	Diphylloside B	Flavonoids	YYH
		C_38_H_48_O_19_	831.2681	−0.2	[M+Na]^+^	121.0269, 299.0541, 355.1166, 547.1760	Diphylloside B	Flavonoids	YYH
29	13.39	C_32_H_38_O_15_	707.2195	0.3	[M+HCOO]^−^, [M−H]^−^, [M+Cl]^−^	146.9657, 353.1024, 514.1468	Epimedoside A	Flavonoids	YYH
		C_32_H_38_O_15_	663.2275	−1.2	[M+H]^+^, [M+Na]^+^	121.0269, 299.0541, 355.1166, 547.1760	Epimedoside A	Flavonoids	YYH
30	13.41	C_26_H_30_O_11_	517.1680	−6.9	[M−H]^−^	219.0671, 327.1270, 383.1110	(2S,3S)-3,5-dihydroxy-2-(4-hydroxyphenyl)-8-(3-methyl-2-buten-1-yl)-4-oxo-3,4-dihydro-2H-chromen-7-yl *β*-D-glucopyranoside	Flavonoids	YYH
31	14.13	C_22_H_22_O_9_	475.1212	−7.0	[M+HCOO]^−^	267.0642, 353.0994	Ononin^*∗*^	Flavonoids	HQ
		C_22_H_22_O_9_	431.1325	−2.8	[M+H]^+^	269.0797	Ononin^*∗*^	Flavonoids	HQ
32	15.81	C_12_H_22_O_6_	301.1069	6.9	[M+K]^+^	167.0713	3-Hexenyl-*β*-glucopyranoside	Saccharide	YYH
33	16.05	C_16_H_12_O_5_	283.0604	−2.9	[M−H]^−^	148.0159, 268.0364	Calycosin^*∗*^	Flavonoids	HQ
		C_16_H_12_O_5_	285.0751	−2.3	[M+K]^+^, [M+Na]^+^	270.0508	Calycosin^*∗*^	Flavonoids	HQ
34	17.76	C_39_H_50_O_19_	883.2882	0.6	[M+HCOO]^−^, [M−H]^−^, [M+Cl]^−^	146.9654, 251.0307, 529.1697, 675.2292	Epimedin A^*∗*^	Flavonoids	YYH
		C_39_H_50_O_19_	839.2961	−0.9	[M+K]^+^, [M+Na]^+^	313.0700, 369.1326, 531.1852	Epimedin A^*∗*^	Flavonoids	YYH
35	18.46	C_38_H_48_O_18_	853.2774	0.2	[M+HCOO]^−^, [M−H]^−^, [M+Cl]^−^	146.9656, 366.1101, 551.1510, 645.2186	Epimedin B^*∗*^	Flavonoids	YYH
		C_38_H_48_O_18_	809.2855	−0.9	[M+H]^+^, [M+K]^+^	313.0698, 369.1322, 531.1849, 677.2419	Epimedin B^*∗*^	Flavonoids	YYH
36	18.88	C_21_H_20_O_6_	369.1322	−2.9	[M+H]^+^	243.0640, 313.0699	Icaritin	Flavonoids	YYH
37	18.93	C_39_H_50_O_19_	867.2924	−0.5	[M+HCOO]^−^, [M−H]^−^, [M+Cl]^−^	146.9660, 366.1099, 551.1497, 659.2339	Epimedin C^*∗*^	Flavonoids	YYH
		C_39_H_50_O_19_	823.3014	−0.6	[M+H]^+^, [M+K]^+^	129.0545, 313.0699, 369.1322, 515.1931, 531.1852	Epimedin C^*∗*^	Flavonoids	YYH
38	18.99	C_22_H_24_O_7_	439.1143	−2.5	[M+K]^+^, [M+Na]^+^	239.0905, 301.0677	3,5,7-Trihydroxy-8-(3-methoxy-3-methylbutyl)-2-(4-methoxyphenyl)-4H-chromen-4-one	Flavonoids	YYH
39	19.17	C_41_H_52_O_21_	881.3114	4.6	[M+H]^+^	85.0294, 135.0437, 243.0645, 313.0698, 369.1321	Hexandraside B	Flavonoids	YYH
		C_41_H_52_O_21_	879.2921	−0.8	[M−H]^−^	267.0300, 367.0621, 451.0672, 513.1100, 613.1210	Hexandraside B	Flavonoids	YYH
40	19.21	C_27_H_30_O_11_	575.1754	−2.9	[M+HCOO]^−^, [M−H]^−^	175.0025, 351.0861, 367.1169, 513.1748	Icariside I	Flavonoids	YYH
		C_27_H_30_O_11_	531.1850	−2.1	[M+H]^+^, [M+Na]^+^	85.0294, 135.0437, 243.0645, 313.0698, 369.1321	Icariside I	Flavonoids	YYH
41	19.22	C_33_H_40_O_15_	721.2337	−1.7	[M+HCOO]^−^, [M+Cl]^−^	175.0025, 281.0437, 367.1169, 451.0672, 513.1748	Icariin^*∗*^	Flavonoids	YYH
		C_33_H_40_O_15_	677.2433	−1.1	[M+H]^+^, [M+K]^+^	135.0437, 313.0698, 369.1321, 531.1851	Icariin^*∗*^	Flavonoids	YYH
42	19.23	C_20_H_24_O_6_	399.1215	2.7	[M+K]^+^	135.0437	Lariciresinol	Phenylpropanoids (lignans)	HQ
43	19.26	C_28_H_24_O_13_	569.1236	−9.4	[M+H]^+^	85.0294, 135.0437, 313.0698, 369.1321	8-Hydroxy-6-methyl-9,10-dioxo-9,10-dihydro-1-anthracenyl 6-O-(3,4,5-trihydroxybenzoyl)-*β*-D-glucopyranoside	Anthraquinones	NX
44	19.63	C_25_H_30_O_8_	481.1785	−9.9	[M+Na]^+^	387.1427	Rubschisantherin	Phenylpropanoids (lignans)	NX
45	20.82	C_36_H_42_O_17_	747.2483	−1.5	[M+H]^+^	299.0532, 355.1114	Korepimedoside A	Flavonoids	YYH
		C_36_H_42_O_17_	745.2332	−2.3	[M−H]^−^	121.0304, 352.0934, 367.1168, 499.1620, 583.1812	Korepimedoside A	Flavonoids	YYH
46	20.94	C_41_H_52_O_21_	879.2892	−4.1	[M−H]^−^	367.1156, 381.0964, 571.1715, 673.2076	Korepimedoside C	Flavonoids	YYH
47	21.01	C_56_H_88_O_25_	1199.5264	1.5	[M+K]^+^	369.1320, 383.1110, 882.4549, 1059.5290	Achyranthoside *D* trimethyl ester	Triterpenoids	NX
48	21.08	C_52_H_84_O_19_	1013.5723	4.3	[M+H]^+^	295.0576, 311.0891, 385.1281, 882.4549	Chikusetsusaponin V butyl ester	Triterpenoids	NX
49	21.09	C_53_H_82_O_25_	1117.5026	−4.1	[M−H]^−^	312.0607, 383.1097, 529.1652, 631.2021, 997.4956	Achyranthoside D	Triterpenoids	NX
50	21.11	C_47_H_74_O_18_	949.4763	−0.5	[M+Na]^+^	295.0576, 311.0891, 882.4549	Chikusetsusaponin IV	Triterpenoids	NX
		C_47_H_74_O_18_	925.4757	−4.9	[M−H]^−^	139.1120, 171.1024, 211.1335, 229.1434, 367.1184	Chikusetsusaponin IV	Triterpenoids	NX
51	21.19	C_48_H_76_O_19_	955.4884	−2.5	[M−H]^−^, [M + HCOO]^+^	352.0934, 367.1168, 631.2021, 793.4236	Ginsenoside Ro∗	Triterpenoids	NX
52	21.24	C_31_H_36_O_14_	631.2021	−1.8	[M−H]^−^	121.0304, 352.0934, 367.1168, 583.1812	Ikarisoside F	Flavonoids	YYH
		C_31_H_36_O_14_	633.2146	−5.0	[M+H]^+^	299.0526, 369.1327, 385.1281	Ikarisoside F	Flavonoids	YYH
53	21.28	C_45_H_60_O_24_	1023.3186	7.8	[M+K]^+^	299.0526, 369.1327, 531.1847, 915.3315	Acuminatoside	Flavonoids	YYH
54	21.31	C_27_H_30_O_11_	529.1669	−8.8	[M−H]^−^	121.0304, 219.0656, 383.1102, 513.1729	Wushanicariin	Flavonoids	YYH
55	21.56	C_35_H_42_O_16_	763.2415	−5.2	[M + HCOO]^−^	367.1184, 381.0975, 555.1833, 645.2117	Sagittatoside C	Flavonoids	YYH
		C_35_H_42_O_16_	719.2591	6.3	[M+H]^+^	313.0706, 369.1324, 383.1134, 385.1288, 531.1848	Sagittatoside C	Flavonoids	YYH
56	21.61	C_46_H_74_O_14_	873.4927	−5.0	[M+Na]^+^	729.3908	Taibaienoside IV	Triterpenoids	NX
57	21.78	C_20_H_18_O_6_	353.1015	−4.3	[M−H]^−^	252.0419	8-Prenylkaempferol	Flavonoids	YYH
		C_20_H_18_O_6_	355.1173	−0.9	[M+H]^+^	253.0480, 269.0803, 299.0550	8-Prenylkaempferol	Flavonoids	YYH
58	21.79	C_16_H_12_O_4_	267.0656	−2.4	[M−H]^−^	252.0419	Formononetin	Flavonoids	HQ
		C_16_H_12_O_4_	269.0803	−2.0	[M+H]^+^, [M+Na]^+^	118.0413, 253.0480	Formononetin	Flavonoids	HQ
59	22.23	C_17_H_16_O_5_	301.1080	3.3	[M+H]^+^	167.0694	(6aR,11aR)-9,10-dimethoxy-6a,11a-dihydro-6H-benzofurano[3,2-c]chromen-3-ol	Flavonoids	HQ
60	22.33	C_47_H_72_O_20_	955.4528	−1.7	[M−H]^−^	382.1079, 645.2166, 835.4451, 925.4768	Achyranthoside C	Triterpenoids	NX
		C_47_H_72_O_20_	995.4167	−8.2	[M+K]^+^	272.2571, 299.0537, 383.1132	Achyranthoside C	Triterpenoids	NX
61	23.03	C_32_H_38_O_14_	645.2190	0.2	[M−H]^−^, [M+Cl]^−^ [M+HCOO]^−^	223.0282, 366.1100	Sagittatoside B	Flavonoids	YYH
		C_32_H_38_O_14_	647.2329	−0.7	[M+H]^+^, [M+Na]^+^	191.0005, 207.0315, 313.0700, 369.1321	Sagittatoside B	Flavonoids	YYH
62	23.54	C_17_H_20_O_6_	343.1163	3.2	[M+Na]^+^	131.0491	1,2-Bis(4-hydroxy-3-methoxyphenyl)-1,3-propanediol	Phenylpropanoids (phenylpropionic acid)	YYH
63	23.89	C_27_H_30_O_10_	513.1762	−0.7	[M−H]^−^	146.9659, 217.0478, 351.0867, 366.1100	Icariside II	Flavonoids	YYH
		C_27_H_30_O_10_	515.1900	−2.2	[M+H]^+^, [M+K]^+^, [M+Na]^+^	135.0434, 243.0638, 313.0698, 369.1322	Icariside II	Flavonoids	YYH
64	23.90	C_21_H_20_O_6_	369.1321	−3.2	[M+H]^+^	135.0434, 243.0638, 313.0698	Anhydroicaritin	Flavonoids	YYH
65	24.53	C_42_H_66_O_14_	793.4358	−2.7	[M−H]^−^, [M+HCOO]^−^	209.0450, 367.1171	Zingibroside R1	Triterpenoids	NX
66	24.58	C_19_H_30_O_9_	437.1603	4.3	[M+Cl]^−^	209.0450	Icariside B3	Terpenoids (Monocyclic monoterpenoids)	YYH
67	25.14	C_41_H_64_O_12_	793.4391	1.5	[M+HCOO]^−^	469.3326, 487.3421	Acetylastragaloside I_qt	Triterpenoids	HQ
68	25.55	C_20_H_18_O_5_	337.1068	−4.0	[M−H]^−^	282.0493	Yinyanghuo D	Flavonoids	YYH
		C_20_H_18_O_5_	339.1227	0.0	[M+H]^+^	283.0583	Yinyanghuo D	Flavonoids	YYH
69	25.84	C_25_H_26_O_6_	421.1666	2.2	[M−H]^−^	351.1189	Yinyanghuo B	Flavonoids	YYH
		C_25_H_26_O_6_	423.1829	6.5	[M+H]^+^	349.1055	Yinyanghuo B	Flavonoids	YYH
70	26.15	C_18_H_32_O_3_	297.2423	−0.3	[M+H]^+^	119.0855	13-Hydroxy-9,11-octadecadienoic acid	Organic acids	HQ
71	26.23	C_41_H_62_O_15_	793.4010	−0.8	[M−H]^−^	631.3795, 673.3972	Achyranthoside C_qt	Triterpenoids	NX
72	26.71	C_45_H_56_O_23_	1003.2895	5.1	[M+K]^+^	313.0709	Korepimedoside B	Flavonoids	YYH
73	26.94	C_27_H_44_O_7_	481.3162	0.4	[M+H]^+^	251.1605	Inokosterone	Steroids	NX
74	28.62	C_16_H_30_O_2_	277.2149	4.1	[M+Na]^+^	223.1695	2,15-Hexadecanedione	Others	YYH
75	29.06	C_25_H_24_O_6_	421.1631	−3.6	[M+H]^+^	367.1135	Yinyanghuo A	Flavonoids	YYH
76	29.63	C_21_H_20_O_5_	351.1228	−2.9	[M−H]^−^	293.0440	Artonin U	Flavonoids	YYH
		C_21_H_20_O_5_	353.1355	−8.2	[M+H]^+^	297.0770	Artonin U	Flavonoids	YYH
77	29.76	C_37_H_60_O_10_	699.3821	−8.5	[M+Cl]^−^	277.2148, 353.2030	AstragalosideII_qt	Triterpenoids	HQ
78	30.20	C_25_H_26_O_5_	405.1700	−1.8	[M−H]^−^	295.0594	8,3′-diprenylapigenin	Flavonoids	YYH
		C_25_H_26_O_5_	407.1848	−1.2	[M+H]^+^	149.0233, 295.0598, 351.1224	8,3′-diprenylapigenin	Flavonoids	YYH
79	32.96	C_16_H_22_O_4_	301.1402	−2.7	[M+Na]^+^, [M+H]^+^, [M+K]^+^	149.0231	DBP	Others	NX
80	37.98	C_30_H_48_O_3_	455.3506	−5.3	[M−H]^−^, [M+HCOO]^−^	277.2164	Oleanolic acid	Triterpenoids	YYH NX
81	38.19	C_18_H_30_O_2_	279.2309	−3.4	[M+H]^+^	95.0854	Linolenic acid	Organic acids	HQ
82	38.31	C_22_H_22_O_11_	507.1170	5.2	[M+HCOO]^−^, [M+Cl]^−^	223.0281	Rhamnocitrin-3-O-glucoside	Flavonoids	HQ
		C_22_H_22_O_11_	501.0842	9.7	[M+K]^+^	191.0004, 207.0315, 281.0502	Rhamnocitrin-3-O-glucoside	Flavonoids	HQ
83	41.16	C_18_H_32_O_2_	281.2470	−1.9	[M+H]^+^	95.0852	EIC	Organic acids	HQ
84	44.22	C_21_H_18_O_11_	447.0959	8.4	[M+H]^+^	191.0001	Baicalin	Flavonoids	NX
85	46.85	C_47_H_78_O_18_	931.5353	9.8	[M+H]^+^	115.0034, 147.0651, 553.3685	Asernestioside A	Triterpenoids	HQ
86	46.86	C_27_H_30_O_16_	655.1574	8.8	[M+HCOO]^−^	91.0223, 223.0270	Rutin	Flavonoids	YYH NX HQ
		C_27_H_30_O_16_	633.1487	9.6	[M+Na]^+^, [M+H]^+^, [M+K]^+^	115.0034, 147.0651, 190.9999, 207.0309, 355.068	Rutin	Flavonoids	YYH NX HQ
87	48.41	C_49_H_80_O_19_	1011.4944	1.8	[M+K]^+^	147.0656, 184.0726, 621.3171	Asernestioside B	Triterpenoids	HQ
88	49.83	C_17_H_14_O_6_	315.0886	7.2	[M+H]^+^	207.0301, 281.0486	Jaranol	Flavonoids	HQ
89	50.62	C_35_H_60_O_6_	621.4377	0.8	[M+HCOO]^−^, [M+Cl]^−^	277.2198	Daucosterol	Steroids	YYH NX
90	52.31	C_33_H_42_O_15_	717.2087	−9.5	[M+K]^+^	221.0835, 429.0876	Wanepimedoside A	Flavonoids	YYH
91	57.66	C_20_H_36_O_2_	309.2763	−8.2	[M+H]^+^	89.0612	Linoelaidyl acetate	Others	YYH
92	57.75	C_29_H_38_O_16_	665.2115	9.5	[M+Na]^+^	133.0854	5′-Hydroxyiso-muronulatol-2′,5′-di-O-glucoside	Saccharide	HQ
93	57.90	C_29_H_48_O	413.3796	4.3	[M+H]^+^	95.0869	Stigmasterol	Steroids	NX

YYH: *Herba Epimedii*; NX: *Radix Achyranthis Bidentatae*; HQ: *Radix Astragali*; ^*∗*^Compared with a reference standard.

**Table 2:** The chemical constituents of the GSL blood prototype identified by UPLC-Q-TOF-MS.

NO.	Observed RT (min)	Formula	Observed m/z	Mass error (ppm)	Adducts	Fragment (+/-)	Identification	Structure class	Origin
1	5.26	C_16_H_18_O_9_	353.0846	−9.2	[M−H]^−^	93.0350	Chlorogenic acid	Phenylpropanoids (phenylpropionic acid)	YYH
2	8.40	C_27_H_44_O_7_	481.3201	8.5	[M+H]^+^	175.1189, 288.2025	Ecdysterone^*∗*^	Steroids	NX
3	12.30	C_43_H_68_O_14_	853.4568	−2.8	[M+HCOO]^−^	137.0231, 312.1296	1-O-{(3*β*,5*ξ*,9*ξ*)-3-[(6-Methyl-*β*-D-glucopyranuronosyl)oxy]-28-oxoolean-12-en-28-yl}-*β*-D-glucopyranose	Triterpenoids	NX
4	12.72	C_38_H_48_O_20_	823.2644	−2.7	[M−H]^−^	214.9992, 312.1294	Diphylloside A	Flavonoids	YYH
5	13.01	C_37_H_46_O_19_	793.2537	−3.0	[M−H]^−^	631.2019	Epimedoside D	Flavonoids	YYH
6	13.34	C_32_H_38_O_15_	661.2083	−8.3	[M−H]^−^, [M+HCOO]^−^	353.0997	Epimedoside A	Flavonoids	YYH
7	17.58	C_39_H_50_O_19_	883.2807	−8.0	[M+HCOO]^−^	675.2247	Epimedin A^*∗*^	Flavonoids	YYH
8	18.44	C_38_H_48_O_18_	853.2745	−3.2	[M+HCOO]^−^, [M+Cl]^−^	252.0406, 366.1081, 645.2177	Epimedin B^*∗*^	Flavonoids	YYH
		C_38_H_48_O_18_	809.2861	−0.2	[M+H]^+^	369.1333, 531.1842	Epimedin B^*∗*^	Flavonoids	YYH
9	18.92	C_39_H_50_O_19_	867.2915	−1.6	[M+HCOO]^−^	366.1059, 659.2331	Epimedin C^*∗*^	Flavonoids	YYH
		C_39_H_50_O_19_	823.3031	1.5	[M+H]^+^	136.0765, 531.1840	Epimedin C^*∗*^	Flavonoids	YYH
10	19.20	C_33_H_40_O_15_	721.2341	−1.2	[M+HCOO]^−^, [M+Cl]^−^	367.1163	Icariin^*∗*^	Flavonoids	YYH
		C_33_H_40_O_15_	677.2413	−4.0	[M+H]^+^	313.0683, 369.1314, 531.1844	Icariin^*∗*^	Flavonoids	YYH
11	19.45	C_25_H_30_O_8_	481.1812	−4.3	[M+Na]^+^	97.0644	Rubschisantherin	Phenylpropanoids (lignans)	NX
12	21.12	C_53_H_82_O_25_	1117.5037	−3.2	[M−H]^−^	217.0817, 365.2313, 413.1985, 585.2846, 785.4175	Achyranthoside D	Triterpenoids	NX
13	21.21	C_48_H_76_O_19_	955.4937	3.0	[M−H]^−^	413.1985, 585.2846, 785.4175	Ginsenoside Ro^*∗*^	Triterpenoids	NX
14	21.92	C_47_H_72_O_20_	955.4622	8.2	[M−H]^−^	353.2213, 405.2627	Achyranthoside C	Triterpenoids	NX
15	22.99	C_32_H_38_O_14_	645.2157	−4.9	[M−H]^−^	223.0270	Sagittatoside B	Flavonoids	YYH
16	23.89	C_27_H_30_O_10_	513.1739	−5.3	[M−H]^−^	366.1082	Icariside II	Flavonoids	YYH
		C_27_H_30_O_10_	515.1908	−0.8	[M+H]^+^	313.0671	Icariside II	Flavonoids	YYH
17	28.61	C_16_H_30_O_2_	277.2140	0.6	[M+Na]^+^	223.1695	2,15-Hexadecanedione	Others	YYH
18	32.96	C_16_H_22_O_4_	301.1382	−9.4	[M+Na]^+^, [M+H]^+^, [M+K]^+^	149.0216	DBP	Others	NX
19	38.18	C_18_H_30_O_2_	279.2323	1.7	[M+H]^+^	95.0865	Linolenic acid	Organic acids	HQ
20	46.60	C_27_H_30_O_16_	645.1256	4.4	[M+Cl]^−^	168.0420	Rutin	Flavonoids	YYH NX HQ
		C_27_H_30_O_16_	633.1444	2.8	[M+Na]^+^, [M+K]^+^	207.0296	Rutin	Flavonoids	YYH NX HQ

YYH: *Herba Epimedii*; NX: *Radix Achyranthis Bidentatae*; HQ: *Radix Astragali*; ^*∗*^Compared with a reference standard.

**Table 3:** Identification of the metabolites from GSL *in vivo*.

NO.	Observed RT (min)	Formula	Observed *m/z*	Mass error (ppm)	Adducts	Identification
M1	4.8	C_39_H_52_O_20_	875.2792	5.2	[M+Cl]^−^	Epimedin C + H_2_ + O
M2	7.4	C_28_H_47_N_3_O_9_S	646.2978	−5.8	[M+HCOO]^−^	Linolenic acid + H_2_O + C_10_H_15_N_3_O_6_S
M3	7.57	C_32_H_55_NO_11_S	696.3213	3.2	[M+Cl]^−^	Ecdysterone + H_2_+H_2_O + C_5_H_7_NO_3_S
M4	7.72	C_27_H_45_O_12_P	591.2631	9.3	[M−H]^−^	Ecdysterone + 2x(+O) + HPO_3_
M5	7.73	C_27_H_47_O_12_P	629.2539	6.4	[M+Cl]^−^	Ecdysterone + O + H_2_O + HPO_3_
M6	7.89	C_24_H_31_O_9_P	493.1677	8.9	[M−H]^−^	Rubschisantherin − COO + HPO_3_
M7	8.24	C_25_H_30_O_10_	535.1793	−5.2	[M+HCOO]^−^	Rubschisantherin + 2x(+O)
M8	8.41	C_37_H_59_N_3_O_15_S	852.3433	8.4	[M+Cl]^−^	Ecdysterone + 2x(+O) + C_10_H_15_N_3_O_6_S
M9	14.8	C_24_H_30_O_7_	429.193	2.7	[M−H]^−^	Rubschisantherin + O – COO
M10	16.62	C_33_H_36_O_21_	803.1385	−7.2	[M+Cl]^−^	Rutin − H_2_O + C_6_H_8_O_6_
M11	19.74	C_39_H_48_O_19_	865.2694	−8.9	[M+HCOO]^−^	Epimedin A − H_2_O
M12	19.79	C_38_H_46_O_20_	821.2456	−6.6	[M−H]^−^	Sagittatoside *B* + C_6_H_8_O_6_
M13	20.03	C_24_H_30_O_6_	413.199	4.9	[M−H]^−^	Rubschisantherin − COO
M14	20.04	C_38_H_46_O_18_	835.2618	−5.8	[M + HCOO]^−^	Epimedin B – H_2_O
M15	22.34	C_27_H_46_O_8_	497.3131	2.2	[M−H]^−^	Ecdysterone + H_2_O
M16	23.09	C_33_H_40_O_14_	659.2317	−4.3	[M−H]^−^	Icariin + H_2_ – H_2_O
M17	24.63	C_32_H_51_NO_12_S	672.312	9.1	[M−H]^−^	Ecdysterone + 2x(+O) + C_5_H_7_NO_3_S
M18	26.05	C_18_H_34_O_4_	313.2376	−2.9	[M−H]^−^	Linolenic acid + 2x(+H_2_O)
M19	27.3	C_18_H_31_O_6_P	409.1519	−8.1	[M+Cl]^−^	Linolenic acid + O + HPO_3_
M20	27.48	C_27_H_49_O_11_P	579.2972	5.5	[M−H]^−^	Ecdysterone + H_2_ + H_2_O + HPO_3_
M21	27.59	C_28_H_49_N_3_O_8_S	586.3145	−4.1	[M−H]^−^	Linolenic acid + 2x(+H_2_) + C_10_H_15_N_3_O_6_S
M22	27.85	C_28_H_47_N_3_O_8_S	630.3034	−5.3	[M+HCOO]^−^	Linolenic acid + H_2_ + C_10_H_15_N_3_O_6_S
M23	27.88	C_18_H_30_O_4_	309.2043	−9.1	[M−H]^−^	Linolenic acid + 2x(+O)
M24	28.66	C_27_H_46_O_7_	527.3212	−2.7	[M+HCOO]^−^	Ecdysterone + H_2_
M25	28.82	C_27_H_44_O_9_	557.2997	5.4	[M+HCOO]^−^	Ecdysterone + 2x(+O)
M26	28.83	C_28_H_47_N_3_O_10_S	616.2894	−2.7	[M−H]^−^	Linolenic acid + O + H_2_O + C_10_H_15_N_3_O_6_S
M27	28.86	C_32_H_55_NO_10_S	680.3184	−8.6	[M+Cl]^−^	Ecdysterone + 2x(+H_2_) + C_5_H_7_NO_3_S
M28	28.97	C_32_H_51_NO_11_S	656.3155	6.9	[M−H]^−^	Ecdysterone + O + C_5_H_7_NO_3_S
M29	29.08	C_27_H_44_O_8_	531.2733	0.5	[M+Cl]^−^	Ecdysterone + O
M30	30.24	C_18_H_32_O_3_	295.2255	−8	[M−H]^−^	Linolenic acid + H_2_O
M31	31.03	C_23_H_41_NO_7_S	520.2627	7.8	[M+HCOO]^−^	Linolenic acid + 2x(+H_2_O) + C_5_H_7_NO_3_S
M32	32.47	C_32_H_47_NO_8_S	640.2711	−0.7	[M+Cl]^−^	Ecdysterone + 2x(−H_2_O) + C_5_H_7_NO_3_S
M33	34.78	C_18_H_32_O_4_	347.1975	−5.8	[M+Cl]^−^	Linolenic acid + O + H_2_O
M34	34.9	C_32_H_49_NO_10_S	638.2981	−3.6	[M−H]^−^	Ecdysterone + O – H_2_O + C_5_H_7_NO_3_S
M35	37.62	C_23_H_30_O_4_	369.2044	−7.4	[M−H]^−^	Rubschisantherin + 2x(-COO)
M36	38.5	C_34_H_45_N_3_O_12_S	754.2396	−3	[M+Cl]^−^	Rubschisantherin-COO + C_10_H_15_N_3_O_6_S
M37	40.72	C_33_H_40_O_17_	753.2191	−7.5	[M+HCOO]^−^	Icariin + 2x(+O)
M38	41.15	C_33_H_52_O_15_	733.3252	−5	[M+HCOO]^−^	Ecdysterone + 2x(+O) + C_6_H_8_O_6_
M39	44.28	C_33_H_56_O_15_	737.3607	0.7	[M+HCOO]^−^	Ecdysterone + 2x(+H_2_O) + C_6_H_8_O_6_
M40	51.66	C_33_H_40_O_23_	803.1956	8.5	[M−H]^−^	Rutin + H_2_O + C_6_H_8_O_6_
M41	53.66	C_39_H_51_O_21_P	921.2341	−1.5	[M+Cl]^−^	Epimedin C + H_2_–H_2_O + HPO_3_
M42	53.67	C_37_H_47_N_3_O_22_S	916.2292	−0.9	[M−H]^−^	Rutin + H_2_ + C_10_H_15_N_3_O_6_S

+H_2_: reduction; +O: oxidation; +H_2_O: hydration; +HPO_3_: phosphorylation; +C_6_H_8_O_6_: glucuronidation; +C_5_H_7_NO_3_S: acetyl cysteine conjugation; +C_10_H_15_N_3_O_6_S: glutathione S-conjugation; –H_2_O: dehydration; –COO: decarboxylation.

## Data Availability

The data used to support the findings of this study are included within the article.

## Supplementary Materials

Supplementary Materials See Table S1 and Figures S1-S5 in Supplementary Materials for comprehensive images and value analysis.
