# Supplementary material for: Identification and Analysis of Chemical Constituents and Rat Serum Metabolites in Gushuling Using UPLC-Q-TOF/MS Coupled with Novel Informatics UNIFI Platform
Source: Evid Based Complement Alternat Med. 2021 Dec 31;2021:2894306. doi: 10.1155/2021/2894306 (PMC8741369; doi:10.1155/2021/2894306)
Supplement: Supplementary Materials — See Table S1 and Figures S1-S5 in Supplementary Materials for comprehensive images and value analysis. [file 2894306.f1.docx]

Supplementary Material

# Supplementary Figures and Tables

## Supplementary Figures

**Supplementary Figure 1.** The total ion chromatography (TIC) of three herbal medicines in positive (A) and negative (B) ion modes. YYH= *Herba Epimedii*, NX= *Radix Achyranthis Bidentatae*, and HQ= *Radix Astragali*.

**Supplementary Figure 2.** UPLC-Q-TOF-MS^E^ analysis of the total ion chromatography (TIC) of the prototype components of GSL into the serum. (A) Positive ion mode; (B) Negative ion mode.

**Supplementary Figure 3.** The mass spectrogram and fragmentation pathway of icariin in positive ion mode.

**Supplementary Figure 4.** The mass spectrogram and fragmentation pathway of ginsenoside Ro in negative ion mode.

**Supplementary Figure 5.** The mass spectrogram and fragmentation pathway of rubschisantherin in positive ion mode.

## Supplementary Table

**Supplementary Table 1.** Constituents of GSL.

| Components | Origin | Amount used (g) |
| --- | --- | --- |
| *Herba Epimedii*  (Yinyanghuo)  (EPIMEDII FOLIUM) | *Epimedium brevicomu Maxim.,*  *Epimedium sagittatum（Sieb. et Zucc.）Maxim.,*  *Epimedium pubescens Maxim.,*  *Epimedium koreanum Nakai* | 15 |
| *Radix Achyranthis Bidentatae*  （Niuxi）  (ACHYRANTHIS BIDENTATAE RADIX) | *Achyranthes bidentata Bl.* | 12 |
| *Radix Astragali*  Huangqi  (ASTRAGALI RADIXi) | *Astragalus membranaceus（Fisch.） Bge.var.mongholicus（Bge.）Hsiao,*  *Astragalus membranaceus（Fisch.）Bge.* | 20 |
| *Concha Ostreae*  Muli  (OSTREAE CONCHA) | *Ostrea gigas Thunberg,*  *Ostrea talienwhanensis Crosse,*  *Ostrea rivularis Gould* | 8 |
